# Supplementary material for: Are carbon emissions trading and green financial instruments synergistic? -Comprehensive quantitative research based on content analysis
Source: PLoS One. 2024 Mar 7;19(3):e0298601. doi: 10.1371/journal.pone.0298601 (PMC10919601; doi:10.1371/journal.pone.0298601)
Supplement: S1 Data — (ZIP) [file pone.0298601.s001.zip › Data/地级市Z_F工作报告.zip!/╣≤╓▌/╫±╥σ╩╨/└┤╫╘╣1⁄2╓┌║┼í╛┬φ┐╦╩2╛▌═°í┐_2008.docx]

各位代表：

现在，我代表市人民政府向大会作工作报告，请予审议，并请各位政协委员提出宝贵意见。

一、2007年工作回顾

2007年，是我市实施“三新一强”战略的起步之年。一年来，在省委、省政府和市委正确领导下，在市人大及其常委会法律监督、工作监督和市政协民主监督下，市人民政府高举中国特色社会主义伟大旗帜，深入贯彻落实科学发展观，按照省委“加快发展黔北综合经济区”和市委“三新一强”战略部署，依托历届党委政府奠定的良好工作基础，承前启后，继往开来，统筹协调，努力推进经济社会又好又快发展，各项事业都取得了新的进步。

（一）经济总量不断壮大，质量效益同步提高

全市生产总值完成566亿元，同比增长14.5%；人均生产总值超过1000美元。三次产业协调发展。一、二、三产业分别增长9.9%、17.7%和13%。投资消费出口同步增加。固定资产投资预计完成259亿元，增长19.6%；社会消费品零售总额完成165亿元，增长18%；进出口总额达到1.5亿美元，增长70.7%。速度质量效益同步提高。财政收入快速增长，完成财政总收入88.59亿元，增长22.65%；完成一般预算收入34.07亿元，增长26.74%。359户规模工业企业赢亏相抵后实现利润57亿元，增长53.8%。

（二）新农村建设扎实推进，农业农村经济发展加快

粮油喜获丰收，粮食总产量330.2万吨，接近历史最好水平;油菜籽产量28.23万吨，增长2.1%。农民人均纯收入2819元，增长10.6%。畜牧业产值完成89.4亿元，增长7.7%；肉类总产量69.26万吨，增长6.9%。收购烟叶149万担。新增“四在农家”创建点1078个，惠及68万农民。“六个一百”工程取得突破性进展，新建沼气池11.3万口，新增优质茶园11万亩，新增药材4.5万亩，新造竹20万亩，种植优质辣椒92万亩，种植商品蔬菜70万亩。农村专业合作组织、农业产业化发展加快，市级龙头企业新增25家，达到78家，实现产值36亿元。“四种工程”有效推进。农村基础设施建设成绩显著，新增农机总动力41万千瓦,实施烟水配套工程41万亩，启动病险水库治理68座，新解决11.5万农民群众的饮水安全问题，东北部4县农村饮水安全工程全面启动；续建和新建通乡油路305公里、通村油路403公里，新建通村公路2507公里，新建乡（镇）客运站32个，启动了农村公路乘客候车亭建设。新增转移农村劳动力14.7万人，成功举办了“中国·正安首届农民工节”。全市第二次农业普查工作全面完成。

（三）新型工业化进程加速，“三个六”工程进展良好

工业经济提速发展。完成规模工业增加值175.45亿元，增长18%，增速提高1个百分点。六大基地、六大标志工程、六大百亿行业（企业）建设取得新进展，十大工业集聚区、白酒“一大十星”培育发展加快，烟酒、机电、化工、能源、新材料、制药等特色优势产业支柱地位进一步巩固。工业项目建设取得重大突破。构皮滩电站建设进展顺利，20万吨竹浆林纸一体化工程进入试运行，遵义县80万吨氧化铝项目、桐梓煤化工项目（一期）、2万吨金属镁项目、遵宝钛业1万吨扩建项目等工程全面开工建设，绥阳煤电化和务正道煤电铝工程项目顺利推进，桐梓火电厂、习水二郎火电厂等项目取得新进展。茅台集团等企业技术改造步伐加快，完成技改投资45亿元。煤炭“两大攻坚战”排难推进，节能减排工作富有成效，规模工业能耗同比下降5.43%，主要污染物减排实现预期目标。

（四）培育创新型城市取得成效，创新活力明显增强

体制机制创新取得新突破。企业“三改一加强”扎实有效，财税、人事、社会保障、土地流转等改革深入推进。投融资改革迈出新步伐，与国家开发银行进行了第二轮合作。诚信遵义建设有效推进，投资软环境治理成效明显，引进到位资金100亿元，增长16.3%。出台实施了《关于鼓励支持和引导个体私营等非公有制经济发展的实施意见》，非公有制经济加快发展。金融体制改革不断深化，金融生态环境建设取得阶段性成果，存贷款稳步增加，存款余额591 亿元，比年初增长18.5%；贷款余额 323亿元,比年初增长11.8%。

科技创新取得新成果。遵义市“十一五”科技发展规划纲要加快实施。国家级、省级企业技术中心新增2家，达到20家；国家级高新技术企业新增5家，达到37家；国家级标准化企业新增1家，达到4家；新增省级产学研合作示范基地1个；企业创新能力增强，拥有自主知识产权的产品不断增加。投入资金3051万元，实施科技项目105个；知识产权保护进一步加强，被列为全国知识产权保护试点城市；专利申报增加，全年申报400件，获得授权300件。国家级、省级新农村科技示范县、乡、村试点获得批准，新材料产业化基地、遵义航天军转民装备制造基地建设扎实推进。

城市建设管理迈上新台阶。城市发展战略研究取得阶段性成果，遵义市城市总体规划修编和新蒲新区规划建设正式启动；完成了10个县城总体规划修编和98个乡（镇）规划编制。城市基础设施建设力度加大，全市城镇化率提高1.5个百分点，达到34.9%。城乡“整脏治乱”全面展开，中心城区交通疏解、环境整治及红花岗公园建设等工作顺利推进，洛江河截污工程全面完成，中心城区供水管网改造完成年度目标。仁怀市、习水县、赤水市和遵义县垃圾、污水处理工程全面竣工。房地产业健康发展，开工建设了20万平方米经济适用住房和4万平方米廉租房。园林绿化、风景名胜区管理、拆除违法建筑等工作取得成效。城市管理水平跃上新台阶，被全国爱卫会命名为“国家卫生城市”，国家级城市名片增加到11张。创建“全国文明城市”、“国家环保模范城市”工作正式启动。围绕打造黔北综合经济区，大力发展快速交通网络和农村公路网络，完成交通投资33亿元。扎南高速公路建成通车；白腊坎至茅台高速、习水至桐梓新站等5条二级公路全面开工，进展顺利。全力推进遵义机场建设，与深圳航空公司达成合作协议。积极协助推进黔渝快速铁路前期工作。开通了遵义至北京、上海、广州三趟始发列车。

（五）文化旅游强市建设有序推进，第三产业加快发展

文化信息共享工程加快实施，新一轮乡（镇）综合文化站建设启动，万村书库、“农家书屋”活动扎实开展，县级“两馆”建设基本完成。市图书馆建成开放，博物馆即将投入使用。数字电视整体平移和“村村通”完成了年度目标，完成转星设施调整67.82万座。文艺创作有效开展，遵义杂技艺术、川剧《红军魂》等文化产品影响不断扩大。非物质文化遗产保护工作加强，27个项目入选省级代表作名录。赤水、习水丹霞地貌“申遗”工作有效推进。娄山关景区、习水“四渡赤水”纪念馆建设等项目如期完成。景区景点等级申报、星级宾馆建设等工作按规划推进，红色旅游商业街——“红军街”正式开放。文化、体育活动广泛开展。成功举办了“激情广场”演出、“放歌十七大，永远跟党走”大型红色经典音乐会和庆祝新中国成立58周年暨撤地设市10周年系列纪念活动。全民健身运动深入开展，竞技体育加快发展，成功举办了“中、美、哈”三国拳击赛、娄山关国际山地运动挑战赛、全省青少年田径锦标赛等赛事和市直机关首届职工运动会，被列为2008·北京奥运会火炬传递城市。以旅游业、新型服务业为主的第三产业快速发展。成功承办了“2007中国·遵义红色旅游可持续发展高峰论坛”,赤水市获“中国优秀旅游城市”称号。全年接待游客782万人（次），实现旅游综合收入55.2亿元，分别增长35.9%和55.4%。大力推进“万村千乡市场”和“新网工程”建设，城乡消费品市场更加活跃。第三产业完成增加值192.6亿元，增长13%。

（六）社会事业全面发展，和谐遵义建设成效明显

民生问题得到高度重视。积极推进就业特别是零就业家庭就业，新增城镇就业人员3.8万人，超计划10.5个百分点；企业从业人员最低工资标准提高100元。社会保障工作不断加强，农村困难群众、城市困难居民实现了应保尽保，保障标准大幅度提高；新型农村合作医疗覆盖全市，参合率达到83.5%；全面启动城镇非从业居民基本医疗保险试点工作；基本养老、基本医疗、失业、生育和工伤保险覆盖面进一步扩大。城市困难居民住房问题得到较好解决，提供了一批经济适用住房和廉租住房。认真做好新阶段扶贫工作，2.44万低收入人口越过温饱线。城镇居民人均可支配收入突破万元，达到11340元，增收2397元，增长18.6%。

教育事业协调发展。农村义务教育“两免一补”全面落实，免除学杂费和书本费1.63亿元，55所农村寄宿制学校、100所农村薄弱学校改造工程全部竣工。“两基”迎“国检”工作扎实推进，荣获国家西部地区“两基”攻坚先进单位，桐梓、道真两县通过省政府“两基”复查和“普实”验收。高中教育加快发展，省级示范性高中新增2所，达到11所。高考成绩实现历史新突破，录取率达55.8%，出台了高中教育质量奖励政策和贫困家庭大学生入学资助政策。职业教育发展加快，招生人数2.76万人。遵义医学院、遵义师范学院本科教学水平顺利通过专家组评估，遵义职业技术学院发展规划通过了专家论证，遵义医药高等专科学校、贵州航天职业技术学院和遵义电大发展加快。

人口和计生等社会事业全面推进。低生育水平继续巩固，人口自然增长率5.13‰；出生人口性别比偏高趋势得到抑制，流动人口管理明显加强，计划生育利益导向机制不断完善，国家人口与计生综合改革试点工作有效推进。基层卫生服务能力增强，55个乡（镇）卫生院建设项目有序推进，其中45个投入使用。城乡公共卫生服务体系、疾病预防控制体系和卫生监督体系进一步健全。“双拥”工作取得显著成绩，连续第六次被评为“全国双拥模范城市”。“十件实事”基本完成。民政、民族宗教、档案、地方志、移民、外事、侨务、妇女、儿童、慈善、老龄人和残疾人等社会事业全面发展。

生态文明建设取得实效。天然林保护、退耕还林工程有序推进，完成造林65万亩，全市森林覆盖率达到44%。农村面源污染治理加强，全市河流水质达标率84.6%，同比提高7.7个百分点。中心城区空气环境质量优良率84.3%，同比提高1.8个百分点。完成了中心城区和各县（市）县城水源保护区域划定工作，市区和所有县城“饮用水源地”水质达标率100%。

“平安遵义”创建深入推进。认真落实“五五”普法规划，扎实推进依法治市和法制宣传工作。切实加强涉法涉诉信访和基层调解工作，各级人民调解组织排查纠纷14169件，调解成功率达98%。化学污染源的监测、监管和食品、药品监督检查及“两网”建设不断加强，打击假冒伪劣产品和非法传销等专项活动取得明显成效。安全生产工作进一步加强，安全生产示范乡（镇）创建活动深入推进，交通、煤矿和非煤矿山等重点领域的安全生产专项整治取得积极成效，安全事故和死亡人数同比分别下降25.65%和11.98%，连续7年实现“双降”。维稳工作和“四民社区”创建扎实推进。突发公共事件应急处理机制进一步完善；社会治安综合治理力度加大，“打防控”机制进一步健全，全市社会安定，人民安居乐业。

（七）政府自身建设不断加强，服务效能明显提高

行政效能进一步提高。市级50个单位561个行政审批项目进入市政务服务中心，共受理行政审批项目19.4万件，办结19.1万件，办结率98.3%，“一站式”服务继续向好。政务公开有效拓展，荣获“全国政务公开先进单位”称号，成为全国政务公开示范点。“数字遵义”扎实推进，“中国遵义”门户网站群运行良好。《遵义市人民政府公报》创刊并免费赠送到村（居），有效地扩大了人民群众的知情权、参与权和监督权。开展了机关作风教育整顿活动，行风评议活动深入推进，群众关心的难点热点问题得到重视和整改。

民主法制和廉政建设全面加强。市政府重大决策专家咨询、重大事项社会公示和听证、新闻发布等制度不断完善。依法行政、行政复议、行政执法监督工作不断加强，政府决策法制化、民主化、科学化水平进一步提高。廉政责任制得到较好落实，审计监督、行政监察和反腐倡廉等工作取得新成果，源头治理力度加大，政风明显好转。自觉接受市人大法律监督、工作监督和市政协民主监督，与各民主党派、工商联和人民团体的联系不断加强。

各位代表，一年来的成绩令人欣慰、催人奋进。这些成绩的取得，是市委统揽全局、科学决策的结果；是市人大、市政协认真监督、积极支持的结果；是全市人民同心同德、努力奋斗的结果。在此，我代表市人民政府，向全市各族人民、广大干部职工，向各民主党派、工商联、人民团体、无党派人士，向驻遵解放军指战员、民兵预备役人员、武警和消防官兵、公安干警，向历届老领导老同志及关心支持遵义经济社会发展的各界人士，表示衷心感谢并致以崇高敬意！

回顾一年来的工作，我们主要有四点体会：一是必须坚持科学发展观，加快转变发展方式，把加快发展与保护生态有机统一起来，大力推进绿色工业、绿色城镇和生态保护协调发展；二是必须坚持解放思想、实事求是、与时俱进，勇于改革创新，敢于攻坚克难，大胆破除制约发展的体制机制障碍；三是必须坚持以人为本，高度关注民生，按照学有所教、劳有所得、病有所医、老有所养、住有所居的要求，不断改善民生；四是必须坚持统筹兼顾，正确处理好整体推进和重点突破、当前和长远的关系，既要切实解决当前最直接、最现实、最紧迫的问题，又要立足长远发展，谋划和推进事关全局的战略性工作。

在肯定成绩的同时，我们也要清醒地看到工作中存在的一些问题和不足：一是在改革的推进上存在不少困难，适应国家宏观调控政策的能力还有待进一步提高；二是安全生产、维稳工作和节能减排形势仍然严峻；三是农村基础设施建设依然薄弱，抵御自然灾害和抗御市场风险的能力不强，产业结构调整的难度大；四是教育、卫生、文化、社会保障等民生问题虽然得到重视，但与人民群众日益增长的物质文化需求仍有差距；五是城市管理长效机制有待进一步完善推进；六是由于食品等价格上涨引起的物价指数上升，对中低收入家庭生活带来影响。我们必须高度重视前进中的各种困难和问题，采取更加有力的措施，切实加以解决。

二、2008年主要工作

2008年是深入贯彻落实党的十七大精神的第一年，是深入实施省委“加快发展黔北综合经济区”战略和市委“三新一强”战略的重要之年，也是“十一五”规划承上启下的一年。做好今年各项工作，对保持我市经济社会又好又快发展十分重要。

指导思想：高举中国特色社会主义伟大旗帜，以党的十七大精神为指导，全面贯彻落实科学发展观，深入贯彻落实省十次党代会、市三次党代会精神，坚持解放思想，坚持改革开放，坚持以人为本，坚持统筹协调，坚持好字优先、能快则快，按照中央和全省经济工作会议部署，继续抢抓发展机遇，创新发展理念，转变发展方式，提高发展质量，更加重视改善民生和促进社会和谐，不断开创经济社会又好又快发展的新局面，全面发展和提升壮大黔北综合经济区。

主要预期目标：生产总值增长12.5%以上；规模工业增加值增长18%；全社会固定资产投资增长20%，确保完成300亿元以上的投资任务；社会消费品零售总额增长16%；财政总收入增长13%以上、超过100亿元，一般预算收入增长13%以上、超过40亿元；城镇居民人均可支配收入增长10%；农民人均纯收入增长8%，超过3000元；城镇登记失业率控制在4.5%以内；人口自增率控制在5.5‰以内；单位生产总值综合能耗下降4.99%，主要污染物排放量减少2%。

围绕以上目标，按照胡锦涛总书记关于“像贵州这样的省区，只要符合科学发展观的要求，有条件、有效益，就要努力加快发展”的要求，扎实抓好十个方面的工作：

（一）以增加农民收入为核心，扎实推进社会主义新农村建设

认真贯彻落实2008年中央1号文件精神，坚持把“三农”工作作为全市工作的重中之重，以促进农民增收为核心，深入推进以“四在农家”创建为载体的社会主义新农村建设。

积极发展现代农业。加大科技增收力度，狠抓“四种”工程，打牢发展基础。高度重视粮、油、烟生产，确保粮食和油菜籽产量稳定在320万吨和28万吨左右，收购烤烟180万担。坚持走高效农业和规模农业发展路子，进一步加快区域产业结构布局调整，实施农业标准化生产，大力发展品牌农业和效益农业，规模推进特色、无公害农产品基地建设，着力打造遵崇、贵遵高速公路和210国道沿线现代农业示范区。围绕“六个一百”工程，新建茶园18万亩以上，集中打造遵义茶叶品牌;新造竹15万亩，种植辣椒100万亩、商品蔬菜80万亩，新种植中药材5万亩。积极培育壮大食用油、优质米、茶叶、辣椒、肉类等农产品龙头加工企业和农产品市场，完成乡镇企业增加值162亿元，确保增长16%。加快生态畜牧业发展步伐，推行“科学养殖、节本增效”养殖模式，大力发展大户养殖、小区养殖和特色养殖，狠抓良种、良繁、防疫等体系建设，提高畜禽优质率、商品率，确保肉类总产量达到80万吨以上。大力发展农村专业合作经济组织，提高农业组织化、专业化、市场化程度，推进现代农业加快发展，促进农村经济全面振兴。

扎实推进新农村建设。坚持不懈地推进“四在农家”创建，全年新增“四在农家”创建点1000个，新增20万户、85万农民受益。加大村庄整治力度，稳步推进农村社区建设，进一步规划建设布局合理、节约耕地、设施完善、风格独具的黔北民居。继续大力抓好沼气池建设，加强后续管理和维护，使更多的农户用上清洁能源。切实加强农业农村基础设施建设，增强农业和农村经济发展活力，大力推进东北部四县农村饮水安全工程进度，全面启动全市农村饮水安全工程，切实解决农村群众饮水安全问题。实施40万亩烟水配套工程，继续抓好遵义灌区、湄凤余灌区二期工程建设，完成68 座病险水库治理，进一步完善水库渠系配套，增强农业防灾抗灾能力。因地制宜推进农业机械化。进一步落实保护耕地的基本国策，严格执行从严控制土地审批的政策，把保护土地资源与加快发展结合起来，抓好土地总规修编和全国第二次土地资源调查。加大土地开发整治力度，确保耕地占补平衡。

大力发展劳务经济。加强劳务输出工作指导，提高劳务输出的组织化程度，培训农村劳动力6.3万人，新增劳务输出10万人以上。积极推进农村科技文化阵地建设，努力实现乡（镇）有综合文化站，行政村有科技文化活动室，有效解决农民看书难和学科技难的问题，加快培养有文化、懂技术、会经营的新型农民。进一步整合“阳光工程”、“雨露计划”、“山海合一”、“企盼行动”项目资源，抓好非农技能培训，发展农村经纪人队伍。认真实施“三关工程”，努力解除外出务工人员后顾之忧。大力推进新阶段扶贫开发，实现易地扶贫搬迁2000人，加大“整村推进”、“集团帮扶”力度，建立健全抑制返贫的长效机制，努力减少低收入人口。

继续深化农村改革。在稳定土地承包的基础上，坚持依法、自愿、有偿原则，积极探索土地流转机制，推进农业规模经营；全面启动并加快集体林权制度改革，深化农技推广、畜牧防疫和水利管理体制等改革；探索乡（镇）机构、农村公益事业建管机制改革，建立健全防止农民负担反弹的长效机制。

（二）以产业结构优化升级为主导，扎实推进新型工业化发展

围绕“高科技、高效益、高就业、低耗能、低污染”目标，以转变发展方式、推进产业升级为主线，以项目建设为突破口，搞好工业规划布局，大力发展循环经济，谋求工业集群的新发展、经济总量的新扩张、产业竞争力的新提升，进一步强化工业经济的主导地位，确保工业经济和各项节能减排指标的实现。

加快工业重大项目发展。扎实抓好项目库建设的各项基础工作，坚持从我市拥有、国家支持的六类特色优势产业项目做起，按照国家产业政策和市场发展需要，切实抓好项目库的充实和更新，抓好主导项目产业链的延伸。进一步抓好烟酒、有色金属、机电、能源、化工、制药等项目建设，大力扶持“一大十星”白酒企业发展，支持做大做强茅台酒集团，振兴遵义名优白酒产业。加快装备制造工业发展，支持江南航天集团、长征电器股份有限公司、贵州海尔电器和天义电器加快产品结构调整。加快发展以中药材深加工为主的地方特色药业，积极推进中药产业化。切实推动构皮滩电站加快建设进度，尽快投产发电。支持遵义县80万吨氧化铝项目抓紧施工，确保第一条生产线建成投产。加快桐梓煤化工一期和桐梓、二郎、绥阳三大煤炭矿区的建设，力争开工建设习水二郎火电厂、桐梓火电厂、绥阳煤电化和务正道煤电铝项目。

着力建设产业集聚区。大力推进工业向园区集中，加快建设遵义循环经济新材料工业集聚区、东北部煤电铝工业集聚区、中心城区装备制造业集聚区、忠庄龙坑南白制药工业集聚区、赤水河谷名酒工业集聚区、东部绿色食品工业集聚区、桐梓循环经济型煤化工工业集聚区、绥阳循环经济型煤电化工业集聚区、赤水循环经济型竹加工工业集聚区。积极开发和推广资源节约、替代和循环利用技术，逐步建立起以循环经济为主导的工业经济体系。

加快重点企业技术改造。大力推进信息化与工业化融合，加快运用信息技术、高新技术和先进适用技术改造提升传统产业步伐。支持遵义航天军转民装备制造等工业企业推进技术改造和技术创新，确保完成茅台酒年新增2000吨技改、贵州华电遵义发电有限责任公司异地技改、遵义钛厂新增1万吨海绵钛技改、遵义铝业环保节能技改、遵义双源化工有限公司异地技改、贵州海尔新增70万台冰箱技改、300万吨干法水泥旋窑生产线技改和白酒“十星”企业技改年度任务，完成技改投资55亿元以上。

强化经济运行协调调度。积极做好煤、电、油、运等资源要素的协调调度，确保经济平稳正常运行，尽力满足人民群众生产生活和经济社会发展需要。加快煤炭工业发展，抓好煤炭资源勘查和道路、电力、供水等基础建设，为煤炭工业快速发展提供条件。加快大矿建设进度，力争容光、花秋二矿、兴隆等规划矿井尽快建成投产。大力推进煤炭资源整合，实现50%的整合煤矿完成扩能技改并投产，全年新增原煤产能400万吨以上。逐步理顺利益关系，切实缓解煤、电供需矛盾，促进煤电互动发展，做大做强能源产业。

（三）以增强城市发展活力为目标，扎实推进创新型城市培育

深入推进改革开放，完善体制机制，提升创新发展能力，增强发展活力，加快培育创新型城市。

深入推进各项改革。充分发挥国有资产管理监督机构职责，推进国有控股企业按照现代企业制度完善公司法人治理结构。大力帮助和推进遵义钛业股份有限公司、贵州航天精工制造有限公司等企业上市；依法推进长征电器集团公司破产，积极配合做好遵义铁合金集团公司、务川汞矿和贵州珍酒厂破产重组工作。认真落实鼓励支持非公有制经济发展的政策措施，促进非公有制经济大发展、大提高；进一步探索公有制经济的多种有效实现形式，通过积极引导法人间相互参股、吸引民间资本入股等多种途径，加快国有企业投资主体多元化进程。建立和完善市与县、县与县之间大项目风险共担和利益分享机制。

努力推动科技进步。深入实施遵义市“十一五”科学技术发展规划纲要，建立完善行业性、区域性科技创新体系，开展好知识产权试点城市工作。以新能源、新材料、电子信息、中药现代化等领域为重点，大力发展高新技术产业。优化提升材料工业，加快遵义国家级新材料产业化基地建设。新建一批企业技术中心，力争建成高新技术企业孵化器。以军转民科技产业示范基地为重点，推动高新技术产业化，打造机电一体化装备制造为主体的产业集群。用足用好税收优惠、财政贴息等政策，加大财政支持力度，引导企业建立科研投入逐步增长的机制，多层次多渠道增加科技创新投入。进一步完善以企业为主体、产学研结合的创新体系，大力扶持一批拥有自主知识产权、自主创新能力、全国全球知名品牌的重点企业，引导企业开展原始创新、集成创新、引进消化吸收再创新，不断开发适应市场需求的新产品。继续认真贯彻落实《全民科学素质行动计划纲要》，不断提高市民的科学文化素质。

加强人才队伍建设。牢固树立人才资源是第一资源的观念，突出抓好专业技术人才、公务员、企业经营管理人才、高级技术人才和农村实用人才队伍建设，推进“人才工程实施，建立“人才强市”的梯次管理体系。加快中小企业各类人才培训力度，着力提升中小企业整体素质。深化事业单位人事制度改革，推进事业单位岗位设置管理和完善收入分配。创新人才工作机制，激发各类人才创造活力和创业激情，形成人尽其才、才尽其用、用当其时的人才工作新局面。

进一步扩大对外开放。认真落实分级负责制，建立招商引资利益分配、成果奖励机制，变被动招商为主动招商，大力促进招商引资。以成渝经济圈、长江经济带、泛珠经济区、友好城市和对口帮扶城市为重点，策划、论证、包装一批以特色农业、新型工业、市政工程、服务业和社会事业为主的大项目和优势项目，集中开展项目推介和招商引资活动，确保招商引资到位资金115亿元以上。进一步营造安商、富商环境，完善项目跟踪服务责任制、重点项目挂帮制，努力提高招商引资项目落户率、资金到位率和开工率。努力扩大对外贸易，着力培育自主出口品牌，优化出口产品结构，继续保持进出口贸易高速增长的良好态势。

（四）以加快第三产业发展为载体，扎实推进文化旅游强市建设

努力促进文化大发展大繁荣。以建立社会主义核心价值体系为目标，加强社会主义精神文明建设，培育文明新风尚，大力开展文明村镇、文明行业、文明机关、文明家庭创建活动。深化文化管理体制改革，积极培育文化市场主体，完善文艺创作和演出体制机制，鼓励和支持重大文艺题材、影视作品创作，推出一批体现先进文化要求、具有市场竞争力的广播影视、新闻出版、文艺精品等文化产品，鼓励发展具有黔北文化特色的文化品牌。建立文化建设财政投入的稳定增长机制。强化文化市场监管和“扫黄打非”工作。认真抓好全国第三次文物普查。抓紧完善海龙屯等文物保护单位的保护利用规划。扎实推进乡（镇）综合文化站、“万村书库”、“农民文化家园”和“农家书屋”试点工程，抓好全国文化信息资源共享试点工程。继续推进有线数字电视整体平移和广播电视“村村通”工作，启动市级广播电视无线覆盖工程。积极筹建遵义市工人文化宫和遵义市档案馆。

做大做强旅游产业。围绕打造文化旅游强市目标，突出“红色”、“绿色”主题，充分利用中国红色旅游可持续发展高峰论坛成果，着力打造红色旅游、生态旅游、乡村旅游、工业旅游、地域文化旅游精品景区、景点，扩大旅游区域合作。推动中国名酒“金三角”国际旅游线路发展，巩固发展广安—重庆—遵义—贵阳红色旅游精品线和重庆—遵义—仁怀—习水—赤水生态、红色旅游并重的精品线，发展赤水河流域旅游联盟和中心城区环城旅游联盟，形成集红色、生态、国酒文化、民族文化和科考探险为一体的旅游带。切实开发利用好三趟始发列车，促进旅游业发展，实现游客1000万人（次）以上，旅游综合收入80亿元以上。完善旅游管理体制，切实整合优势旅游资源，加大重点景区市级统筹管理和推进力度；加快高星级酒店等旅游基础设施和景区配套设施建设，加快推进旅游景区质量等级、全国工农业旅游示范点、星级饭店、乡村旅馆申报评定工作。继续推进赤水、习水丹霞地貌“申遗”工作，着力创建赤水国家级生态示范市。

大力发展新型服务业。加快特色专业批发市场、物流中心建设，形成以城区为中心、集镇为依托、辐射城乡、方便快捷的商贸服务中心和特色农产品集散地，推进城区商场、城郊市场和农村“万村千乡市场工程”、“新网工程”建设，逐步构建现代物流体系。积极发展连锁经营、物流配送、电子商务等服务业，大力促进信息通讯、中介服务、医疗保健、体育健身等新型服务业发展。大力开发、包装、推介旅游商品，做大做强与旅游相关的行、住、食、购、娱等服务行业。

千方百计扩大消费。努力提高城乡居民收入水平，倡导科学、节约、健康的消费方式，引导居民转变消费观念，扩大即期消费。保障市场供应，加强价格监管，有效防止物价过快上涨。进一步整顿和规范市场经济秩序，以食品、农资、药品等为重点，深入开展打击制售假冒伪劣商品等专项活动，促进城乡居民放心消费、扩大消费。

（五）以加快城市建设为重点，扎实推进城乡经济社会协调发展

按照省委、省政府实施大中城市带动战略的要求和构建遵义200万城市人口框架目标，依托中部、西部和东部区域不同资源禀赋、区位条件、产业基础、资源环境承载能力和发展潜力，通过生产力布局、产业结构调整、资金支持等措施，促进中部区域率先发展，做优做强；促进西部经济带尽快崛起，发展更快更好；促进东北部各县发挥后发优势，提速提档跃进，最终形成东部后来居上、中部率先跨越和西部加速发展的新格局，逐步实现城市、城镇强劲带动，打造黔北城市群，在工业化、信息化、市场化和国际化方面发挥区域引领作用，为全面发展和提升壮大黔北综合经济区提供强有力的城市经济支撑。

高起点规划城市。按照“南北拓展、东扩西控、优化中心、完善功能、突出特色”的基本思路，做好城乡规划工作。完成遵义市城市总体规划大纲修编、新蒲新区控规编制、遵义机场航空港区详规编制工作，重点抓好董公寺片区、南白—龙坑片区、遵义大道片区、高桥片区等重点区域的规划编制，推进各县、区（市）加快城镇规划编制工作。完成湘江河景观规划修编和中心城区山体绿化、水面保护规划的编制，启动遵义市城市综合交通规划修编工作。

高标准建设城市。按照“打造提升老城，开发建设新区”要求，紧紧抓住与国家开发银行第二轮合作的机遇，完成中心城区市政工程建设投资12亿元以上。加快推进老城区市政道路建设改造，完成中心城区停车场站（点）规划建设，不断完善城市路网，有效疏解城市交通。开工并着力推进北海路金融一条街、遵义会议接待中心和遵义海关等项目建设。加快推进中桥水库相关工作。实施中心城区污水、垃圾处理及环境整治工程，加强市政基础设施和环保监测能力建设，完善城市功能。全面完成万里路片区改造还房工程，加快推进万里路中心商贸区开发建设，打造城市结点。加快城中村和棚户区改造工作，启动南门关省冶建片区、北京路长征八厂片区、天义厂片区、高桥入城口片区、新舟坝片区旧城改造。以快速干道建设为切入点，抓好新蒲新区开发建设，重点抓好新区市政道路、市政基础设施、给水和污水处理等工程建设。继续抓好汇川区董公寺片区，红花岗区东出口片区、南部新城片区和遵义县南白—龙坑片区开发建设。大力推进强县强镇建设，切实抓好以县城、建制镇为重点的小城镇建设，完成小城镇建设投资26亿元以上。有效推进户籍制度改革，促进城镇化发展，力争城镇化率提高1.5个百分点。加强茅台镇环境整治，培育、发展一批特色乡（镇），充分发挥重点集镇在区域中的辐射带动作用。加大对房地产市场整治和调控力度，促进房地产业健康发展，完成房地产投资42亿元以上。加快经济适用住房和廉租房建设，全面实施城市低收入家庭住房保障制度，基本实现应保尽保。

高效能管理城市。以创建全国文明城市、国家环保模范城市和“整脏治乱”为载体，加强城市综合管理，切实巩固“创卫”成果。深入推进“数字化、人性化城管”、交通秩序整治、环境综合整治和园林绿化风景名胜区管理等工作。建立和完善城市管理长效机制，营造“整洁、有序、文明、安定”的城镇环境。支持各县（市）创建国家级、省级卫生县城和省市级卫生乡（镇），提高城镇管理水平，不断改善人居环境。

（六）以实施交通引领经济为方向，扎实推进交通基础设施建设

抢抓西部大开发和贵广快速铁路、高速公路建设机遇，大力推进公路、机场、铁路、航运建设，逐步形成立体交通网络，为实施大中城市带动战略、推进县域经济发展提供高速快捷路网支持。全力抓好高速公路、二级公路建设，确保遵义至茅台高速公路、习水至新站等5条二级公路建成通车。积极创造条件，力争开工建设机场高速、仁赤高速、杭瑞高速（遵义段）公路。积极推动黔北高速公路、桐梓新站至绥阳太白、南白至尚嵇二级公路建设前期工作。继续推进乡际间油路、通村油路和通村公路建设。积极推进进寨公路、乡（镇）汽车场（站）建设。坚持建管并重，完善县、乡、村公路管护机制，提高公路通达能力。加快遵义机场启用和建设有关工作。积极协调做好黔渝快速铁路前期工作。加强乌江、赤水河流域航道整治，扎实推进码头、船闸规划建设，提高通行能力。

（七）以建立公共财政体系为主线，扎实推进财税金融工作

高度重视财源建设。充分运用税收优惠、财政贴息等政策，大力发展新兴后续财源，壮大提升骨干财源，努力培植财源新增长点，不断增强财政实力。切实加强收支管理。认真做好重点行业、企业等骨干税源的征管和监控工作，依法应收尽收，确保主体税源稳定增长。调整财政支出结构，逐步建立起公共财政框架，进一步增加民生、社会事业投入，严格控制一般性支出，适度集中财力办大事。切实加强扶贫、退耕还林等方面专项资金的监管，确保各类资金足额及时到位、发挥最大效益。加强政府债务管理，切实偿还和化解政府债务。深化财税金融改革。继续抓好农村税费、政府收支分类、部门预算、政府采购、农村金融等改革，完善市县财政管理体制和利益分享机制。做好金融工作，改善金融生态环境建设，加强银政、银企合作，培育担保体系，争取各金融机构加大对重点项目、支农项目、中小企业等方面的信贷支持，努力缩小存贷差，实现金融与地方经济共同发展。

（八）以全面发展社会事业为着力点，扎实推进和谐遵义建设

按照“学有所教、劳有所得、病有所医、老有所养、住有所居”的整体要求，扎实推进各项工作。

倍加关注民生。以解决人民最关心、最直接、最现实的利益问题为重点，切实加强就业、社会保障等工作，使经济发展成果更多体现到改善民生上。完善就业和创业服务体系，加快建立城乡统一的人力资源市场，拓宽就业渠道，新增城镇就业人员3.5万人，实现下岗失业人员再就业0.8万人，使经济发展与扩大就业相协调。切实维护企业、员工合法权益，防止拖欠企业职工工资和民工工资，构建和谐劳动关系。重视社会公平，合理调节收入分配，不断增加城乡居民收入。以创建“四在农家”、“四民社区”为载体，努力改善群众生产生活条件。逐步建立健全与经济发展水平相适应、覆盖城乡的多层次社会保障体系，建立完善社会保障长效机制，做到保障对象应保尽保，保障资金应发尽发。完善新型农村合作医疗和城镇居民基本医疗制度，确保城镇居民基本医疗保险参保率达到85%以上、新型农村合作医疗参合率达到90%以上。完善社会保障制度，扩大社会保障覆盖面，不断提高保障水平；推进企业职工养老保险和失业保险市级统筹、农民工医疗保险、农民工工伤保险、民选村居干部养老保险工作，继续抓好养老保险扩面工作，强化养老保险基金征缴，提高养老保险基金的支撑能力。完成2000户农村茅草房危房改造工程。大力发展慈善事业，建成市儿童福利院和市流浪未成年人救助保护中心，改善老年公寓和敬老院的生活条件，让人民群众共享改革发展新成果。

全面发展社会事业。优先发展教育。大力发展义务教育，继续巩固“两基”攻坚成果，下大力气提升“两基”和“普实”水平，确保“两基”顺利通过“国检”。加快发展高中阶段教育，有效增加普通高中教育资源。大力发展职业教育，建立健全职业教育经费保障机制。支持遵义职业技术学院和县级职业教育中心建设，确保遵义职业技术学院顺利通过教育部人才培养工作评估验收。着力提高高等教育质量，推进高等教育资源优化整合，积极做好遵义大学筹建工作，积极申报遵义干部学院。重视学前教育、成人教育、特殊教育，支持民办教育发展。努力加大投入，增加中心城区基础教育资源，进一步缓解城区入学矛盾。扎实抓好各类教育工程。加大薄弱学校改造力度。认真做好资助贫困家庭学生工作。坚持计划生育基本国策，统筹解决人口问题，稳定低生育水平；抓好国家人口与计生综合改革试点工作，继续治理出生人口性别比，提高流动人口管理水平；深化计生奖励扶助机制，积极探索建立农村计划生育家庭养老保障制度。坚持公共医疗卫生的公益性质，坚持预防为主、以农村为重点、中西医并重，建立健全公共卫生服务体系、医疗服务体系、医疗保障体系和药品供应保障体系；着力构建农村三级卫生服务网络和城市社区卫生服务体系，加强乡（镇）卫生院建设。扶持中医药和民族医药事业发展。认真落实《全民健身计划纲要》，广泛开展群众性体育活动；积极承办各类赛事，精心组织北京奥运火炬在遵传递活动。认真做好统计工作，按时完成全国第二次经济普查任务。完成遵义市地方志编纂初稿。加强妇女、儿童和未成年人工作，切实保障妇女、儿童合法权益，有效预防未成年人犯罪。以促进各民族共同进步、繁荣发展为主题，积极推进民族事业发展。积极创建全国残疾人无障碍示范城市，大力发展残疾人事业。进一步做好“双拥”工作，巩固发展“全国双拥模范城市”创建成果。加强全民国防教育，推动全民国防建设。认真做好民政、移民、老年人、人防、外事、侨务、宗教、气象、水文、档案、地方志等工作，推进各项社会事业全面发展。

加强生态文明建设。切实保护青山绿水，加强工业污染综合治理，大力推进节能减排，为建立资源节约型社会和环境友好型社会奠定良好基础。建立健全节能减排与环境保护统计、监测、考核体系，完善节能降耗减排目标考核制度，认真执行“一票否决”制。高度重视城区空气质量的监测监管，按照国家产业政策和淘汰落后产能时间表，按期关闭高耗能、高污染企业，确保完成节能减排任务。继续抓好天然林工程、退耕还林工程建设，加快推进300里竹廊等工程建设；加快推进高速公路、二级公路沿线绿化工作，打造景观走廊；抓好森林防火和病虫害防治工作；力争森林覆盖率提高1个百分点。认真抓好污染源普查。继续治理农村面源污染，重点抓好饮用水源区和重点流域环境综合整治。高度重视地质灾害、石漠化综合治理，全面推进农村生态家园建设。

全力维护社会稳定。扎实抓好“五五”普法，全面提升依法治市水平。切实加强法律服务和法律援助工作，保护人民群众合法权益。丰富民主形式，拓宽民主渠道，保障人民的知情权、参与权、表达权和监督权。加快村（居）民自治进程，完成第七次村居换届工作。扎实推进“平安遵义”创建，建立城乡打防控一体化机制，健全社会治安防控体系；加大社区、学校、企业周边和“城中村”环境整治力度，依法严厉打击各类违法犯罪活动，确保人民群众安居乐业。加强药品食品监管和农村“两网”建设，加强食品、药品安全保障。完善各类应急预案，健全公共突发事件应急机制，提高处理公共突发事件的应对能力。加强信访和基层调解工作，切实化解民间纠纷，畅通民意诉求渠道，切实维护好人民群众的切身利益，努力把不和谐因素化解在萌芽状态，维护社会稳定。强化安全生产“一票否决”责任制，认真开展“安全生产隐患治理年”活动，切实做到关口前移、重心下移，加强道路交通、煤矿、非煤矿山、烟花爆竹、消防及危险化学品等重点行业的安全管理，坚决遏制重特大事故发生，实现安全事故持续“双降”。

（九）以解决群众切身利益为根本，扎实推进“十件实事”完成

1.建设乡际间油路和通村油路200公里，建成和改造通村公路2000公里，新增178个行政村通公路；2.建成农村沼气池12万口；3.解决农村70万人饮水安全问题；4.完成18万平方米经济适用住房、2万平方米廉租房；5.完成100所农村义务教育阶段薄弱学校改造工程；6.建设遵义市市民体育健身中心，建成80个村级农民体育健身工程；7.新建广播电视“村村通”工程3000座；8.建成忠庄城市公共交通始发站和14个港湾式候车亭，新增中心城区500个公共停车位，建成北海路等5条城市主干次道，改造东风桥、万里桥；9.启动中心城区垃圾无害化处理二期工程、董公寺污水处理工程和颜村污水处理厂改扩建，完成高坪河污水截流工程以及中心城区15个垃圾中转站建设；10.建设遵义市文化艺术中心。

（十）以提高行政效能为中心，扎实推进政府自身建设

进一步加强学习。坚持一手抓适应，一手抓提高。大力弘扬理论联系实际的马克思主义学风，把学习贯彻落实十七大精神与抓好本地区本部门的实际工作结合起来，围绕建设学习型政府、学习型机关的目标，加大各级各类干部培训力度，不断拓宽视野，完善知识结构，提高政治素质和业务水平，增强工作本领。

切实推进依法行政。坚持科学、民主决策，继续推行专家咨询制度、决策责任制度、重大事项社会公示和听证制度，重要会议邀请市人大、市政协参加制度，规范性文件报人大常委会备案与审查制度，自觉接受市人大及其常委会法律监督、工作监督和市政协民主监督。完善公众参与、专家论证和政府决策相结合的决策机制，建立健全行政执法责任制、过错责任追究制、行政赔偿制和评议考核机制，切实规范行政审批，严格按照法定权限和程序行使职权、履行职责，推进各级政府民主、科学、依法行政。建立健全决策权、执行权和监督权既相互制约又相互协调的权力结构和运行机制。进一步完善和执行好政府采购、招投标等各项工作制度。继续推进政务、厂务、村务公开，实行收费“明白卡”制度，切实整治各种乱收费，推进“阳光行政”，增强政府工作透明度和公信力；坚持用制度管权、管事、管人，加强对权力运行的监督和制约，加强对重点部门领导和重点岗位人员人财物权力的监管。坚持标本兼治、综合治理，惩防并举、注重预防的方针，建立健全教育、制度、监督并重的惩治和预防腐败体系，深入落实党风廉政建设责任制，坚持不懈地开展反腐倡廉工作。加强审计监督和行政监察，抓好行政监察网建设，推进源头治理、标本兼治。

加快转变政府职能。全面履行政府经济调节、市场监管、社会管理和公共服务的职能，坚持政府保公平，市场管效率，充分发挥市场对资源的基础性配置作用，将政府更多的精力和资源用在加强社会管理、提高公共服务水平上。切实加强政府效能建设，巩固作风教育整顿成果，继续深入开展行风评议活动，大力改进会风和文风，严格控制各种检查、评比、达标活动，降低行政成本。继续推进“数字遵义”建设，加快信息化进程，充分整合现有行政办公网络资源，逐步形成政府业务协同办理和网上服务的工作格局，努力提高政府工作效率。坚持分级负责原则，保护和调动各地加快发展的积极性、主动性和创造性。严格执行投资“五定”制度，建立健全重点项目领导挂帮责任制度，完善岗位责任制度、考核制度和报告制度，加大督查和行政问责力度，提高政府执行力，确保各项工作按计划均衡推进。

各位代表，自今年1月中旬至2月初，我市14个县、区（市）均遭受了50年来最严重的雪凝灾害，给广大人民群众的生产生活和工农业生产造成巨大损失。面对严重的灾情，我们高度重视，及早安排部署，积极妥善应对，各级干部身先士卒，各方面力量和广大人民群众积极投身参与，大力发扬团结一致，克服困难，英勇奋斗，众志成城，情系民生的精神，全力以赴抢险救灾，努力把灾害造成的损失降到最低程度。通过上上下下的艰苦努力，取得了阶段性胜利，并涌现出了许许多多可歌可泣、感人致深的事迹。灾害给我们造成的损失是巨大的，灾后重建和发展的任务十分艰巨。我们一定要以抗灾救灾保经济社会发展，以灾后重建促经济社会发展，更好更快地推进“三新一强”和黔北综合经济区建设。一是要认真总结和吸取这次雪凝灾害的经验教训，切实增强防灾抗灾能力；二是要切实关注民生，妥善安排好受灾群众和其他困难群众的生产生活，确保不出现非正常情况；三是要加大投入，全力抓好电力、交通、供水、水利、通讯等基础设施的恢复重建，加强工作（生产）调度，强化安全生产管理，保证生产生活需要；四是要抓住农时，切实做好春耕备耕工作，确保农业不减产、农民要增收；五是要切实抓好市场供应，加强物价监管，依法打击违法行为，切实维护正常的生产生活秩序；六是要抓好受损景区、景点的修复和旅游宣传推介工作，积极筹备组织好茶叶博览会、饮食文化博览会和竹文化节等活动，推进第三产业快速发展；七是要切实加强雨雪冰冻次生灾害的防范应对工作，确保预防到位、处置及时、应对有力；八是加强领导，落实责任，为夺取灾后重建和发展新胜利提供坚强的组织保证。我们坚信，有省委、省政府和市委的坚强领导，有各方面的大力支持配合，有全市各族人民群众的共同奋斗，我们有信心、有决心、有能力继续把抢险救灾和灾后重建与发展的各项工作做好，一定能够把灾害造成的损失减少到最低程度，实现今年经济社会发展的各项目标任务。

各位代表！时代赋予我们重任，人民寄予我们厚望，遵义已站在由总体小康向全面小康迈进的新起点上。让我们紧密团结在以胡锦涛同志为总书记的党中央周围，高举中国特色社会主义伟大旗帜，以中国特色社会主义理论为指导，深入贯彻落实科学发展观，在市委正确领导下，聚精会神搞建设，一心一意谋发展，开拓创新促跨越，为建设小康遵义、和谐遵义而努力奋斗！
